# Integrated Metabolomic and Transcriptomic Analyses Reveal Alterations in the Serotonergic Synapse Pathway and a Robust Diagnostic Model in Ulcerative Colitis

**DOI:** 10.3390/metabo16040263

**Published:** 2026-04-14

**Authors:** Haiyan Wang, Hanlin Wu, Yuzhen Fu, Xuhan Lv, Chao Li, Yan Jin, Wei Ge, Zenan Wu

**Affiliations:** 1 Formula-Pattern Research Center, Jiangxi University of Chinese Medicine, Nanchang 330004, China; 2 Department of Postgraduate, Jiangxi University of Chinese Medicine, Nanchang 330004, China; 3 Department of Anorectal Surgery, Affiliated Hospital of Jiangxi University of Chinese Medicine, Nanchang 330006, China; 4 School of Clinical Medicine, Jiangxi University of Chinese Medicine, Nanchang 330004, China

**Keywords:** ulcerative colitis, metabolomic, transcriptomic, serotonergic synapse pathway, diagnostic model

## Abstract

Objectives: To overcome the limitations of invasive diagnostic approaches for ulcerative colitis (UC) diagnosis, this study integrates liquid chromatography–mass spectrometry (LC–MS)-based serum metabolomics with mucosal transcriptomics to elucidate the interplay between systemic metabolic perturbations and neuroendocrine signaling in UC pathogenesis. Methods: Serum metabolites and mucosal differentially expressed genes (DEGs) were identified through multi-omics profiling. Key neurotransmitter receptor-related genes (NRRGs) were prioritized using three machine learning algorithms: LASSO, Random Forest, and SVM-RFE. A three-gene diagnostic nomogram was developed and rigorously validated across multiple independent cohorts (GSE48958, GSE73661) using receiver operating characteristic (ROC) curve analysis and decision curve analysis (DCA). Results: The integrated analysis revealed 334 dysregulated metabolites and 3093 DEGs, both converging on the serotonergic synapse pathway. Specific molecular alterations were uncovered, including tryptophan depletion linked to the downregulation of SLC6A4, concomitant with abnormal serotonin accumulation and PTGS2-mediated inflammatory responses. The three-gene signature, HTR3C, RPS6KA6, and NETO2, formed a highly robust diagnostic model, achieving an area under the ROC curve (AUC) exceeding 0.96 in both the training cohort and external validation sets. Conclusions: This multi-omics study delineates a neuroimmune mechanism in UC centered on dysregulation of the serotonergic synapse. The resulting three-gene nomogram identifies a candidate biomarker signature that demonstrates strong discriminative potential; however, given the exceptionally high performance metrics, these findings should be interpreted as a preliminary diagnostic framework rather than a clinically validated tool, and its efficacy relative to standard markers like CRP or fecal calprotectin requires further investigation in prospective real-world cohorts. Nonetheless, this study provides critical mechanistic insights into gut–brain axis dysfunction in UC.

## 1. Introduction

Ulcerative colitis (UC) is a chronic, relapsing form of inflammatory bowel disease characterized by continuous mucosal inflammation that begins in the rectum and extends proximally through the colon [1]. The epidemiology of UC is undergoing a marked shift, transforming from a condition historically concentrated in Western countries into a global health concern. Notably, incidence rates are rising rapidly in newly industrialized regions, driven by urbanization and environmental changes [2]. Despite significant advances in therapeutic approaches, including the introduction of biologic agents, clinicians continue to face substantial challenges in accurately diagnosing UC and monitoring disease activity over time [3]. Colonoscopy with histopathological evaluation remains the gold standard for both the diagnosis and assessment of mucosal healing; however, its invasiveness, high cost, and procedure risks severely restrict its utility for repeated monitoring [4]. Meanwhile, widely used non-invasive biomarkers such as C-reactive protein (CRP) and fecal calprotectin (FC) demonstrate important limitations [5]. CRP exhibits low sensitivity for localized colonic inflammation, while FC suffers from high inter-individual variability and limited specificity, often failing to reliably differentiate UC from other gastrointestinal conditions, including colonic polyps and infectious enteropathies [6,7]. These shortcomings underscore an urgent need for novel, robust, and non-invasive biomarkers that more faithfully capture the complex molecular pathology underlying UC.

The pathogenesis of UC involves complex interactions among epithelial barrier dysfunction, dysregulated immune responses, and intestinal microbiota imbalance [8,9,10]. In recent years, the gut–brain axis has emerged as a critical regulator of intestinal homeostasis [11]. Neurotransmitters traditionally thought to act exclusively in the central nervous system, such as dopamine and norepinephrine, are also synthesized in the gut, where they serve as key signaling molecules that modulate immune cell activity and maintain epithelial integrity [12,13]. Together with serotonin, these neurotransmitters form intricate neuroendocrine signaling networks that influence both local intestinal function and systemic immune responses, processes highly relevant to UC pathogenesis [14]. However, the diagnostic utility of neurotransmitter receptor-related genes (NRRGs) in UC, along with the systemic metabolic features underlying neuroimmune crosstalk, remains poorly explored [15,16].

At the molecular level, a persistent inflammatory microenvironment drives metabolic adaptation and functional reprogramming in intestinal epithelial cells and multiple immune cell populations, thereby sustaining chronic inflammatory [17,18]. Transcriptomic studies show that the dysregulation of energy metabolism and mitochondrial-related pathways in UC mucosa is closely linked to disease severity and response to therapy [19]. Clinical evidence further indicates that the expression of glycolytic rate-limiting enzyme hexokinase 2 (HK2) correlates with the degree of inflammation, implicating metabolic reprogramming in disease progression [20]. Metabolomic analyses have also revealed marked differences in serum metabolic profiles between patients with UC and healthy controls, allowing reliable distinction not only between UC and healthy individuals but also between UC and Crohn’s disease (CD) [21]. Moreover, integrating metabolomic data with machine learning approaches has demonstrated potential for refining the classification of UC phenotypes, including disease extent [22].

Given the complexity of the underlying biological networks, single-omics approaches often yield fragmented insights into disease mechanisms [23,24]. Integrating serum metabolomics, which reflects systemic functional states, with tissue transcriptomics, which captures local pathogenic processes, offers a powerful multi-omics strategy for uncovering metabolic–transcriptional crosstalk in UC [25,26]. However, the high dimensionality and inherent noise of biomedical datasets present significant challenges for traditional statistical methods, which are susceptible to multicollinearity and overfitting [27,28]. Machine learning algorithms, owing to their strong capacity for pattern recognition and feature selection, have been widely used in biomarker discovery for complex diseases [29]. Yet individual algorithms are limited by their specific methodological assumptions. To improve biomarker robustness and model generalizability, integrated multi-algorithm feature selection strategies have gained increasing traction. Despite these advances, the systematic role of the neuro-immune-metabolic axis remains under-explored. Specifically, this study addresses this knowledge gap by uniquely focusing on neurotransmitter receptor-related genes (NRRGs). Unlike prior incremental multi-omics approaches [21], we sought to determine whether systemic metabolic signatures of the gut–brain axis consistently align with local mucosal receptor reprogramming, providing a more targeted mechanistic framework for noninvasive diagnostics.

**Figure 1:**
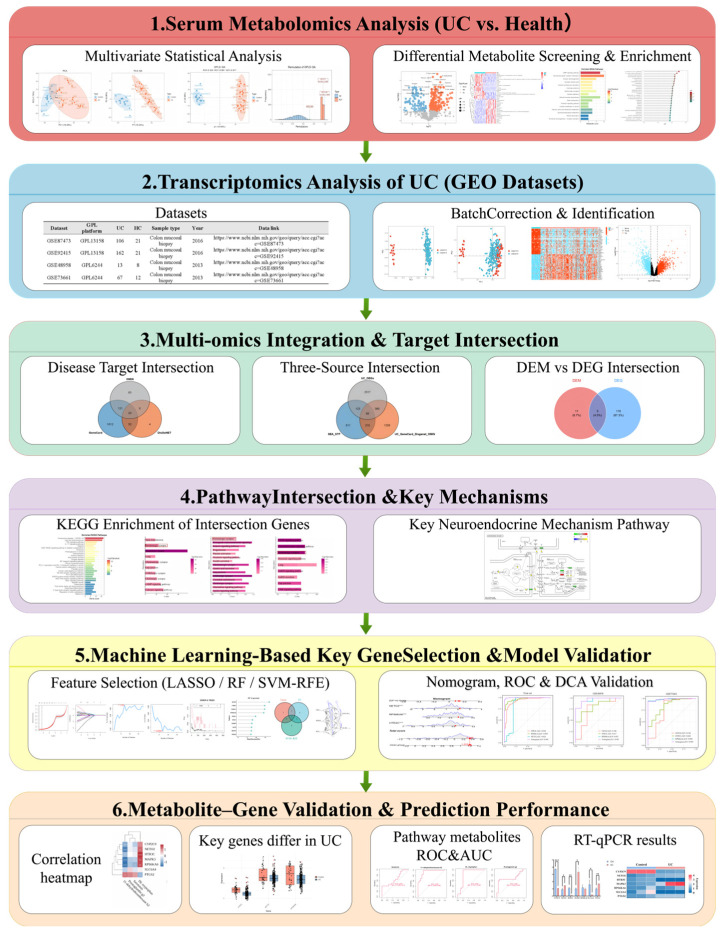
Schematic overview of the integrated multi-omics and machine learning workflow for key gene identification. (1) Differential analysis of serum metabolomic profiles. (2) Differential expression analysis and batch effect correction of GEO transcriptomic datasets. (3) Multi-omics intersection analysis to identify candidate target genes. (4) Pathway intersection analysis to identify core perturbed mechanisms. (5) Machine learning-based feature selection, followed by construction and validation of a nomogram diagnostic model. (6) Gene–metabolite correlation analysis and assessment of discriminative performance.

In this study, we implemented an integrative multi-omics framework that combines LC–MS-based serum metabolomic profiling from a clinical cohort with large-scale mucosal transcriptomic datasets for UC obtained from the Gene Expression Omnibus (GEO). Our goals were to characterize perturbations in the serotonergic synapse pathway and to elucidate the relationship between systemic metabolic alterations and colonic gene expression. Furthermore, by applying multiple machine learning algorithms, including LASSO, random forest, and SVM-RFE, we systematically identified key features among NRRG. Using NRRGs as the candidate feature space, we then developed an interpretable multigene nomogram for UC diagnosis and evaluated its robustness and potential clinical utility in independent external cohorts. A schematic overview of the study workflow is presented in Figure 1.

## 2. Materials and Methods

### 2.1. Data Sources

Serum metabolomic data were generated from human serum samples analyzed by ultra-performance liquid chromatography coupled with quadrupole time-of-flight mass spectrometry (UPLC–QTOF–MS). Bulk transcriptomic microarray datasets derived from colonic mucosal biopsy samples of patients with UC and healthy controls, specifically GSE87473, GSE92415, GSE48958, and GSE73661, were obtained from the GEO database (https://www.ncbi.nlm.nih.gov/geo/ (accessed on 1 December 2025)). To ensure cohort comparability, clinical metadata including age, disease severity, and treatment status were extracted from the original series matrix files. Detailed information on these datasets is summarized in Table 1, which highlights significant clinical heterogeneity in terms of disease severity (ranging from limited to extensive colitis) and treatment history (including pre-treatment screening and post-therapy samples). Although sex information was not provided in the original GEO submissions for GSE87473 and GSE92415, this absence represents a critical metadata gap given the potential for sex-specific differences in systemic metabolism. Both cohorts consist of patients with well-defined UC phenotypes at baseline. Putative target genes of differentially abundant serum metabolites were predicted using the SwissTargetPrediction (STP) database (http://www.swisstargetprediction.ch/) and the Similarity Ensemble Approach (SEA) database (https://sea.bkslab.org/). UC-associated disease targets were collected from the GeneCards database (https://www.genecards.org/), the DisGeNET database (https://www.disgenet.org/), and the Online Mendelian Inheritance in Man (OMIM) database (https://omim.org/), using the search terms “ulcerative colitis” or “UC”. Neurotransmitter receptor–related gene (NRRG) sets were curated by integrating gene lists from the supplementary materials of two published studies [30,31] and by querying the Molecular Signatures Database (MSigDB, GSEA; https://www.gsea-msigdb.org/) with the keyword “neurotransmitter receptor”. A total of 205 genes were initially retrieved from MSigDB. After combining all three sources and removing duplicates, a final set of 448 NRRGs was obtained, as detailed in Supplementary Table S1.

### 2.2. LC–MS-Based Serum Metabolomic Analysis

#### 2.2.1. Study Population and Sample Collection

All study participants were recruited from the Affiliated Hospital of Jiangxi University of Chinese Medicine. UC was diagnosed in accordance with the American College of Gastroenterology (ACG) clinical guidelines for adult UC, based on a combination of clinical manifestations, colonoscopic findings, and the histopathological evaluation of colonic mucosal biopsies, after excluding infectious and other forms of colitis. The control group comprised age-matched healthy individuals who underwent routine physical examinations during the same period and were confirmed to be free of inflammatory bowel disease and other organic intestinal disorders. Although the serum cohort and the GEO transcriptomic cohorts are biologically comparable in their focus on UC versus healthy controls, differences in disease stage, activity, and treatment status may influence tryptophan metabolism and serotonergic signaling. This potential confounding factor is acknowledged as a limitation. Inclusion criteria were as follows: (1) age between 18 and 70 years, regardless of sex; (2) fulfillment of established diagnostic criteria for UC and availability of complete clinical data for participants in the UC group; (3) absence of clinically apparent abnormalities on physical examination and routine auxiliary tests in the control group; and (4) provision of written informed consent. The exclusion criteria included (1) the presence of severe cardiovascular, hepatic, pulmonary, renal, or hematopoietic diseases; (2) gastrointestinal malignancies, intestinal tuberculosis, or a history of intestinal surgery; (3) severe complications such as intestinal obstruction, intestinal perforation, or toxic megacolon; and (4) pregnancy or lactation. Peripheral venous blood samples (3 mL) were collected from all participants after an overnight fast. Samples were allowed to clot at room temperature for 30 min and then centrifuged at 4000 rpm for 10 min at 4 °C. The resulting serum supernatant was carefully aspirated, aliquoted, and immediately stored at −80 °C until metabolomic analysis. This study was approved by the Medical Ethics Committee of the Affiliated Hospital of Jiangxi University of Chinese Medicine (Approval No. JZFYLL20231020036). Written informed consent was obtained from all participants prior to enrollment. Baseline demographic and clinical characteristics, including age, sex, body mass index (BMI), disease duration, and Mayo score, are summarized in Table 2.

#### 2.2.2. LC–MS Analysis

Serum metabolites were separated on an Agilent 1290 Infinity UHPLC system (Agilent Technologies, Santa Clara, CA, USA) using a hydrophilic interaction liquid chromatography (HILIC) column (2.1 mm × 100 mm, 1.7 μm). The column temperature was maintained at 25 °C, with a flow rate of 0.5 mL/min and an injection volume of 2 μL. The mobile phase consisted of solvent A, water containing 25 mM ammonium acetate and 25 mM ammonium hydroxide, and solvent B, acetonitrile. The gradient elution program was as follows: 95% B from 0 to 0.5 min, a linear decrease from 95% to 65% B between 0.5 and 7 min, a further linear decrease from 65% to 40% B from 7 to 8 min, maintenance at 40% B from 8 to 9 min, a rapid increase back to 95% B from 9 to 9.1 min, and re-equilibration at 95% B from 9.1 to 12 min. Samples were kept at 4 °C in the autosampler and injected in randomized order. Quality control (QC) samples were interspersed at regular intervals to monitor instrument stability and ensure data reproducibility. Following chromatographic separation, mass spectrometric detection was carried out on a TripleTOF 6600 mass spectrometer (SCIEX, Framingham, MA, USA) operating in both positive and negative electrospray ionization (ESI) modes. Ion source parameters were set as follows: Gas 1, 60 psi; Gas 2, 60 psi; curtain gas (CUR), 30 psi; source temperature, 600 °C; and ion spray voltage, +5500 V (positive mode) or −5500 V (negative mode). The MS scan range was *m*/*z* 60–1000 with an accumulation time of 0.20 s, and the MS/MS scan range was m/z 25–1000 with an accumulation time of 0.05 s. Data-dependent acquisition (IDA) mode was used for MS/MS analysis, with precursor ions automatically selected based on signal intensity for fragmentation. The declustering potential (DP) was set to +60 V (positive mode) or −60 V (negative mode), and the collision energy (CE) was 35 ± 15 eV. IDA settings included a dynamic exclusion window of 4 Da and acquisition of up to 10 MS/MS spectra per cycle.

#### 2.2.3. Metabolomic Data Preprocessing and Differential Metabolite Identification

Raw metabolomic data were preprocessed as follows: first, they were normalized by total ion current (TIC) to correct for instrument variability; second, Pareto scaling was applied to reduce the dominance of high-intensity peaks; and third, missing values affecting fewer than 5% of features were imputed using k-nearest neighbors (k = 5). Principal component analysis (PCA) was performed using the prcomp function in R to evaluate overall data structure and analytical stability, with 20 samples per group included in this assessment. Of the initial 60 samples, 30 from the UC group and 30 from the healthy control group, 10 per group were deliberately reserved for independent RT-qPCR validation of key genes; the remaining 20 per group underwent LC–MS metabolomic profiling. A small number of additional samples were excluded during quality control due to technical variability. Partial least squares discriminant analysis (PLS-DA) and orthogonal partial least squares discriminant analysis (OPLS-DA) models were constructed using the ropls R package 4.5.2. To assess model robustness and guard against overfitting, permutation tests were performed with 100 iterations for PLS-DA and 1000 iterations for OPLS-DA. Differential metabolites were identified using the following criteria: variable importance in projection (VIP) > 1, |log_2_ fold change (FC)| ≥ 0.585, and false discovery rate (FDR) < 0.05. Log_2_FC values were derived from independent two-sample *t*-tests. The threshold of VIP > 1 was selected to prioritize metabolites with significant contributions to group separation in the OPLS-DA model, while the $|\log_{2}FC|\geq 0.585$ cutoff (equivalent to a 1.5-fold change) was employed to ensure that the identified perturbations were biologically substantial and less likely to be influenced by analytical noise. Metabolites annotated with Kyoto Encyclopedia of Genes and Genomes (KEGG) identifiers were subjected to pathway annotation, and KEGG pathway enrichment analysis was conducted using the enricher function in the clusterProfiler R package, with the Benjamini–Hochberg procedure for FDR correction consistently applied across all enrichment analyses.

### 2.3. Transcriptomic Analysis

Colonic mucosal gene expression matrices for GSE87473, GSE92415, GSE48958, and GSE73661 were downloaded from the GEO database. Among these, GSE87473 and GSE92415 were combined to form the training set, while GSE48958 and GSE73661 served as independent external validation cohorts. In the training set, duplicated probes were collapsed using the avereps function, and genes with a mean expression value of zero across all samples were removed. Only genes common to both datasets were retained and merged into a single, unified expression matrix. To maintain analytical consistency across disparate datasets, all transcriptomic data underwent a standardized preprocessing pipeline including log2 transformation and quantile normalization. Batch effects were corrected using the ComBat algorithm implemented in the sva R package, with tissue type included as a covariate in the model matrix. For samples with missing clinical metadata (e.g., sex), they were analyzed based on their verified disease status, and the lack of these covariates was factored into the discussion of model limitations. PCA was performed both before and after batch correction to assess the effectiveness of this adjustment. Differential gene expression analysis was carried out using a standardized linear modeling framework, with FDR correction applied via the Benjamini–Hochberg procedure consistently across all relevant analyses.

### 2.4. Feature Selection, Model Construction and Validation

#### 2.4.1. Machine Learning-Based Identification of Key NRRGs

Three machine learning approaches were applied to identify key NRRGs exclusively within the training set (the integrated GSE87473 and GSE92415 datasets) to prevent data leakage. First, least absolute shrinkage and selection operator (LASSO) regression was implemented for feature selection using a binomial logistic regression model (α = 1). Ten-fold cross-validation was used to select the optimal regularization parameter (λ), and genes with non-zero coefficients at this λ value were retained. Second, a random forest model was built using the randomForest R package with 500 trees (ntree = 500) and 10 variables randomly sampled as candidates at each split (mtry = 10). Gene importance was assessed using the Mean Decrease Gini index, and the top 10 genes were selected based on this metric. Third, support vector machine–recursive feature elimination (SVM-RFE) was performed using the caret, kernlab, and e1071 R packages. A five-fold cross-validation strategy combined with recursive feature elimination was applied to identify the gene subset yielding the lowest classification error rate. Crucially, the external validation cohorts (GSE48958 and GSE73661) remained entirely independent and were not used during the feature selection and model training phases. The final set of key NRRGs was defined as the intersection of genes selected by all three methods within the training domain only. All scaling and normalization parameters were estimated solely from the training set and then applied to the validation data to ensure a strict separation between model development and performance assessment. Scaling and normalization parameters were estimated solely from the training set and then independently applied to the external validation cohorts (GSE48958 and GSE73661). Although nested cross-validation was not employed in this study, the use of independent external validation cohorts supports the generalizability of the model. Future work may incorporate nested cross-validation to further mitigate overfitting risks.

#### 2.4.2. Construction and Validation of the Three-Gene Nomogram Model

A multivariable logistic regression model was developed using the machine learning-selected key genes to construct a diagnostic nomogram. Model fitting was carried out with the lrm function in the rms R package, and calibration was assessed via bootstrap resampling (1000 iterations). Receiver operating characteristic (ROC) curve analysis was performed using the pROC package with 1000 bootstrap replicates to estimate 95% confidence intervals for the area under the curve (AUC). Sensitivity, specificity, positive predictive value (PPV), and negative predictive value (NPV) were calculated at the optimal cutoff determined by the Youden index. Discriminative performance was evaluated using ROC analysis, and clinical utility was assessed through decision curve analysis (DCA). Following development in the training set, the nomogram was independently validated in the GSE48958 and GSE73661 cohorts. Model performance in both validation cohorts was quantified using the AUC and net benefit.

#### 2.4.3. Forest Plot Analysis of Candidate Gene Risk Associations

Multivariable logistic regression analyses were conducted to evaluate the associations between candidate genes and UC status. Forest plots were generated using the forestplot R package, with an odds ratio (OR) of 1 serving as the reference line. Age-adjusted ORs and their corresponding 95% confidence intervals are displayed on a logarithmic scale, accompanied by associated *p* values.

#### 2.4.4. Artificial Neural Network Construction

An artificial neural network (ANN) classification model was developed using differentially expressed NRRGs as a complementary exploratory tool to capture potential non-linear relationships among NRRG features that may not be discernible under the linear assumptions of logistic regression. The expression values of upregulated and downregulated genes were dichotomized at the median to create binary input features. These binarized NRRG features served as input variables, and sample group labels (UC vs. control) were used as the output variable. A single-hidden-layer ANN with five neurons in the hidden layer was implemented using the neuralnet R package. Model performance was assessed via confusion matrix analysis.

### 2.5. Integrated Multi-Omics Analysis

#### 2.5.1. Multi-Omics Pathway Intersection and KEGG Mapping

UC-associated gene sets were compiled by integrating data from the OMIM, GeneCards, and DisGeNET databases. Differential metabolites identified in the metabolomic analysis were subjected to target prediction using the Similarity Ensemble Approach (SEA) and SwissTargetPrediction databases. Only human target genes were retained, merged, and deduplicated to generate a metabolite-associated gene set. The UC-associated gene set, transcriptomic differentially expressed genes, and metabolite target gene set were then intersected, yielding 93 key target genes. These genes were analyzed for KEGG pathway enrichment using the clusterProfiler R package, with gene identifiers converted to Entrez IDs and mapped to human pathway annotations. FDR correction (Benjamini–Hochberg procedure) was consistently applied across all enrichment analyses. To identify shared pathway-level alterations between metabolomics and transcriptomics, significantly enriched KEGG pathway IDs derived from differential metabolites and key target genes were extracted and intersected. Venn diagrams were generated using the ggvenn R package to visualize overlapping pathways. Differential genes and metabolites associated with these shared pathways were subsequently mapped onto KEGG pathway diagrams for integrative visualization.

#### 2.5.2. Identification of Key Pathways and Gene–Metabolite Correlation Analysis

Key pathways were identified by comparing KEGG enrichment results across three gene sets: the metabolite target gene set, the UC differentially expressed gene set, and the UC-associated disease gene set. Pathways significantly enriched in all three sets were defined as key pathways. Differentially expressed genes linked to these key pathways were extracted from KEGG annotations and integrated with the machine learning-dentified key genes. KEGG-annotated differential metabolites associated with the same pathways were also included for downstream integrative analyses. Group-wise differences in gene expression and metabolite abundance were evaluated using two-group comparisons and visualized accordingly. Gene–metabolite correlations were assessed using Spearman’s rank correlation coefficients and displayed as clustered heatmaps. *p* values were adjusted for multiple testing using the Benjamini–Hochberg FDR method. To evaluate the association between candidate genes and UC status, univariate logistic regression was performed to estimate ORs and 95% confidence intervals, which were visualized in forest plots. ROC curve analysis was used to assess the discriminative performance of individual genes and metabolites; AUC values and their 95% confidence intervals were calculated using the DeLong method.

### 2.6. RT-qPCR Experiment

For experimental validation, whole blood was diluted 1:1 with phosphate-buffered saline (PBS) and gently mixed. The mixture was carefully layered onto Ficoll-Paque™ separation medium along the tube wall and centrifuged at 450× *g* for 25 min at room temperature. The peripheral blood mononuclear cell (PBMC) layer was collected, resuspended in PBS, and centrifuged again at 250× *g* for 10 min. The supernatant was discarded, and the resulting cell pellet was retained for subsequent experiments. Total RNA was extracted using a column-based kit following the manufacturer’s instructions, treated with DNase to remove genomic DNA contamination, and eluted in RNase-free water (stored on ice for short-term use or at −80 °C for long-term storage). Complementary DNA (cDNA) was synthesized in a 20 μL reaction using a reverse transcription kit (37 °C for 15 min, followed by enzyme inactivation at 85 °C for 5 s). Quantitative real-time PCR was then carried out in a 20 μL reaction containing 10 μL SYBR qPCR Mix, 2 μL cDNA, 0.4 μL each of the forward and reverse primers, and nuclease-free water to final volume, using a two-step amplification protocol. GAPDH served as the internal control, and relative gene expression levels were calculated using the 2^−ΔΔCt^ method. Primer sequences are provided in Supplementary Table S2.

### 2.7. Statistical Analysis

All statistical analyses were conducted using R software (version 4.4.1). A two-sided *p* value < 0.05 was considered statistically significant (* *p* < 0.05, ** *p* < 0.01, *** *p* < 0.001). Continuous variables are reported as mean ± standard deviation or as median with interquartile range (IQR), depending on the distribution of the data, and were compared between groups using Welch’s *t*-test. Categorical variables were analyzed using Pearson’s chi-square test or Fisher’s exact test, as appropriate. Differential metabolites were identified by integrating variable VIP scores, fold change magnitude, and results from multiple testing correction. Differential gene expression analysis of transcriptomic data was performed using a standardized linear modeling framework with FDR correction applied via the Benjamini–Hochberg procedure. Correlation analyses were carried out using Spearman’s rank correlation coefficient. Given the significant age difference between groups, all logistic regression analyses examining gene–UC associations and those used for nomogram construction were implemented as multivariable models with age included as a covariate. Associations between candidate genes and UC status were assessed using age-adjusted logistic regression, and results are presented as ORs with corresponding 95% confidence intervals. Discriminative performance was evaluated using ROC curve analysis; the AUC and its 95% confidence interval were estimated using the DeLong method. DCA was performed to assess the net clinical benefit of the diagnostic model across a range of threshold probabilities.

## 3. Results

### 3.1. Global Serum Metabolomic Differences and Multivariate Statistical Analysis

**Figure 2:**
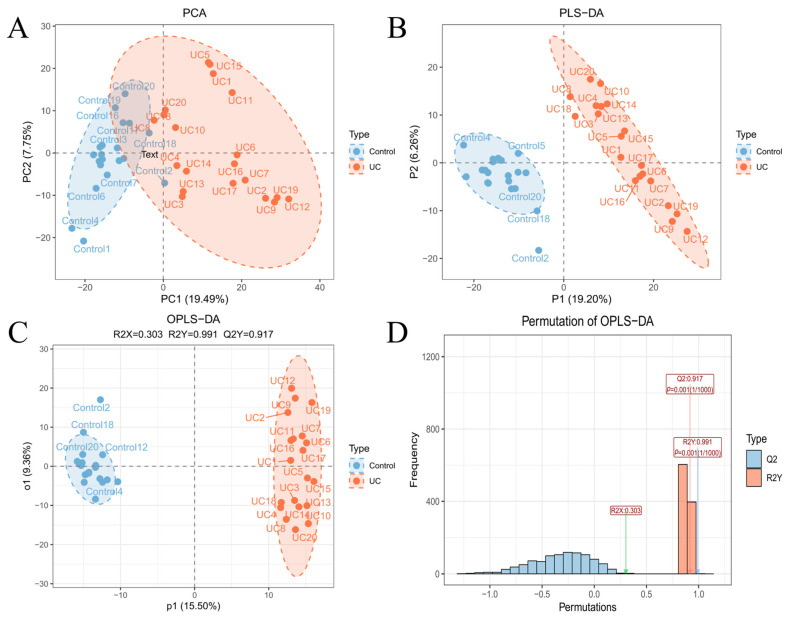
Multivariate statistical analysis of serum metabolic profiles in the control and UC groups. (**A**) PCA score plot. (**B**) PLS-DA score plot. (**C**) OPLS-DA score plot. (**D**) Permutation test results for the OPLS-DA model.

Multivariate statistical analyses were conducted on serum samples from patients with UC and healthy controls. PCA revealed a clear separation trend between the two groups in the score plot, primarily along the first principal component (PC1 = 19.49%, PC2 = 7.75%) (Figure 2A). PLS-DA further enhanced this group separation (P1 = 19.20%, P2 = 6.26%) (Figure 2B). OPLS-DA yielded a distinct separation between UC and control samples along the predictive component p1 (p1 = 15.50%, o1 = 9.36%) (Figure 2C), with strong model fit and predictive performance (R^2^X = 0.303, R^2^Y = 0.991, Q^2^ = 0.917). Permutation testing (1000 iterations) confirmed that the R^2^Y and Q^2^ values of the original model were substantially higher than those obtained from permuted models (*p* = 0.001, based on 1/1000 permutations), demonstrating robust model validity with no indication of overfitting (Figure 2D). Together, these findings indicate pronounced global alterations in serum metabolic profiles between patients with UC and healthy controls.

### 3.2. Identification of Differential Metabolites and Metabolic Pathway Enrichment Analysis

**Figure 3:**
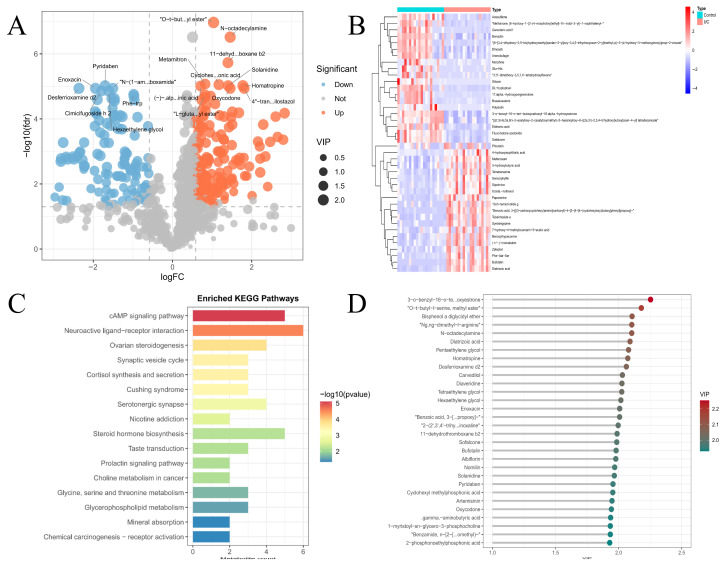
Differential metabolite analysis and pathway enrichment. (**A**) Volcano plot of differential metabolites. (**B**) Heatmap and hierarchical clustering of differential metabolites. (**C**) Bar plot of KEGG pathway enrichment analysis. (**D**) Score plot of high-VIP metabolites.

Differential metabolites were identified using the OPLS-DA model based on the following criteria: variable VIP > 1, |log_2_ FC| ≥ 0.585, and FDR < 0.05, resulting in 334 differential metabolites (Figure 3A). The top 20 most discriminating metabolites, ranked by VIP score, fold change magnitude, and adjusted *p* value, are listed in Supplementary Table S3. Hierarchical clustering heatmap analysis showed that these differential metabolites clearly distinguished UC samples from healthy controls (Figure 3B). KEGG pathway enrichment analysis revealed 16 significantly enriched pathways (*p* < 0.05), with differential metabolites primarily associated with signal transduction and neuroendocrine-related processes. Among these, the cAMP signaling pathway showed the highest enrichment significance and included five metabolites, while the neuroactive ligand–receptor interaction pathway contained the largest number of metabolites (six) (Figure 3C). To further prioritize high-impact metabolites, a more stringent threshold of VIP > 1.9 was applied, yielding 30 candidate metabolites with strong discriminative power; the highest VIP score reached 2.2 (Figure 3D).

### 3.3. Integration of Transcriptomic Datasets and Differential Gene Expression Analysis

**Figure 4:**
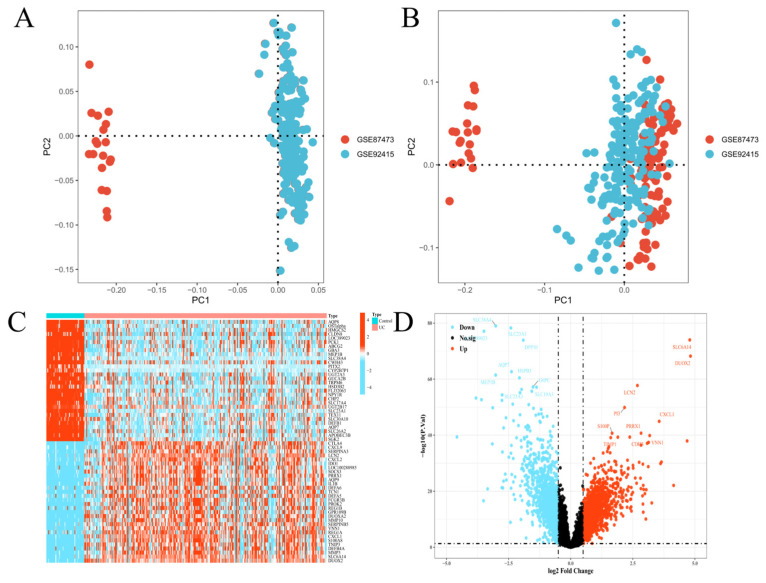
Batch effect correction and differential gene expression analysis of UC transcriptomic datasets. (**A**) PCA score plot before batch effect correction. (**B**) PCA score plot after ComBat batch correction. (**C**) Heatmap and hierarchical clustering of DEGs. (**D**) Volcano plot of DEGs.

The training datasets GSE87473 and GSE92415 were integrated for transcriptomic analysis, and batch effects were systematically assessed. PCA initially showed that samples clustered primarily by dataset origin, indicating substantial batch effects (Figure 4A). Following correction with the ComBat algorithm, samples from both cohorts became well intermixed in the PC1–PC2 space, confirming the effective removal of batch-related variation (Figure 4B). Differential expression analysis identified a total of 3093 differentially expressed genes (DEGs), including 713 upregulated and 2380 downregulated genes. The top 20 DEGs, ranked by adjusted *p* value and fold change, are listed in Supplementary Table S3. Hierarchical clustering heatmap analysis revealed consistent and distinct expression patterns of DEGs between UC and control samples (Figure 4C). A volcano plot further illustrated the global distribution of differential expression, with significantly upregulated and downregulated genes clearly separated on opposite sides of the plot (Figure 4D).

### 3.4. Identification of Key NRRG-Related DEGs Using Multiple Machine Learning Approaches

**Figure 5:**
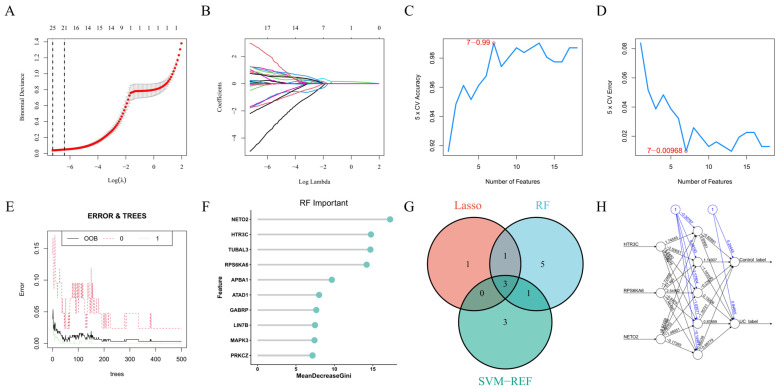
Identification of key NRRG-related DEGs using multiple machine learning approaches. (**A**) Ten-fold cross-validation results for the LASSO regression model. (**B**) Coefficient profile plot from the LASSO regression. (**C**) Five-fold cross-validation classification accuracy across varying numbers of features in the SVM-RFE analysis. (**D**) Classification error rate across varying numbers of features in the SVM-RFE analysis. (**E**) Relationship between the number of decision trees and the OOB error rate in the random forest model. (**F**) Feature importance ranking from the random forest model, displaying the top 10 most important genes. (**G**) Venn diagram showing the overlap of genes selected by LASSO, random forest, and SVM-RFE. (**H**) Schematic representation of the ANN model built using the selected key genes.

To identify NRRG-related DEGs with the highest diagnostic potential, three machine learning-based feature selection methods, LASSO, random forest (RF), and SVM-RFE, were applied. In the LASSO regression model, the optimal regularization parameter (λ) was selected via 10-fold cross-validation (Figure 5A). The coefficient profile plot showed the progressive shrinkage of gene coefficients toward zero as λ increased (Figure 5B). SVM-RFE was implemented using 5-fold cross-validation combined with a recursive feature elimination strategy. Classification accuracy initially rose and then declined as the number of features was reduced (Figure 5C), with the lowest classification error observed at an optimal feature subset (Figure 5D). For the RF model, 500 decision trees were constructed, and the out-of-bag (OOB) error rate steadily decreased and plateaued as the number of trees increased (Figure 5E). Gene importance was ranked using the Mean Decrease Gini index, yielding the top 10 genes: NETO2, HTR3C, TUBAL3, RPS6KA6, APBA1, ATAD1, GABRP, LIN7B, MAPK3, and PRKCZ (Figure 5F). Integration of the three methods revealed a set of overlapping genes, visualized in a Venn diagram (Figure 5G). Ultimately, HTR3C, RPS6KA6, and NETO2 were consistently selected by all three algorithms and were designated as key NRRG-related DEGs. Their consistent identification across distinct algorithmic frameworks underscores their stable discriminative value and provides a robust foundation for downstream mechanistic analyses. Additionally, an ANN classification model built on NRRG-related DEGs successfully classified samples. The network architecture clearly illustrated the weighted connections among the input layer (NRRG gene features), a single hidden layer (five neurons), and the output layer (control vs. UC groups) (Figure 5H). The model generated probability estimates for each sample’s group assignment. Confusion matrix evaluation demonstrated strong classification performance in both groups, confirming the ANN’s ability to effectively distinguish UC from control samples. Overall, the NRRG-based feature set achieved high predictive accuracy (sensitivity = 0.92, specificity = 0.89, AUC = 0.95). Although it did not surpass the performance of the nomogram model, this finding supports the latter as the primary diagnostic tool.

### 3.5. Construction and Validation of the Key Gene-Based Nomogram Model

**Figure 6:**
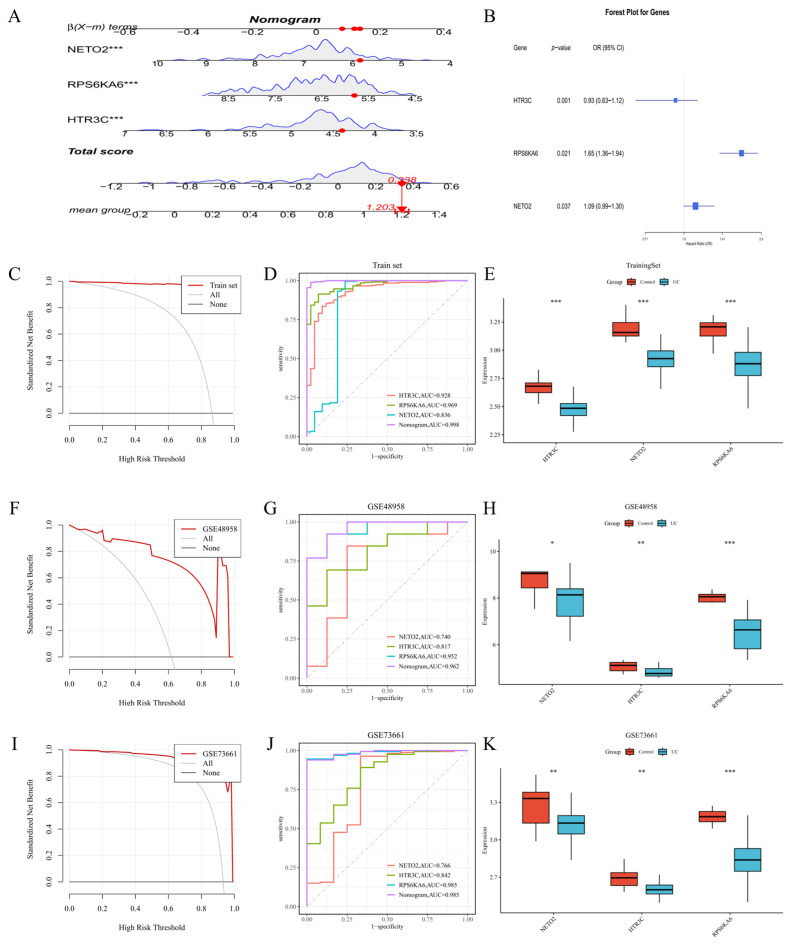
Risk assessment of key genes and construction and validation of the nomogram diagnostic model. (**A**) Diagnostic nomogram based on HTR3C, RPS6KA6, and NETO2. (**B**) Forest plot showing odds ratios (ORs) for the three key genes. (**C**) DCA of the nomogram in the training set. (**D**) ROC curves for the nomogram and individual genes in the training set. (**E**) Expression distributions of the three key genes across UC and control groups in the training set. (**F**) DCA of the nomogram in the GSE48958 validation cohort. (**G**) ROC curves for the nomogram and individual genes in the GSE48958 cohort. (**H**) Expression distributions of the three key genes in the GSE48958 cohort. (**I**) DCA of the nomogram in the GSE73661 validation cohort. (**J**) ROC curves for the nomogram and individual genes in the GSE73661 cohort. (**K**) Expression distributions of the three key genes in the GSE73661 cohort. * *p* < 0.05, ** *p* < 0.01, *** *p* < 0.001.

A diagnostic nomogram was developed using the three key genes, HTR3C, RPS6KA6, and NETO2, as predictors (Figure 6A). Age-adjusted multivariable logistic regression, visualized via a forest plot, revealed that RPS6KA6 (OR = 1.65, 95% CI: 1.36–1.94) and NETO2 (OR = 1.09, 95% CI: 0.99–1.30) were positively associated with UC risk, suggesting potential roles as risk factors, whereas HTR3C showed a negative association (OR = 0.93, 95% CI: 0.63–1.12), indicative of a possible protective effect (Figure 6B). These associations remained robust after adjustment for age. In the training set, all three genes exhibited significant differential expression between UC patients and healthy controls (Figure 6E). ROC curve analysis demonstrated excellent discriminative performance for the nomogram, with an AUC of 0.998 (90% CI: 0.995–1.000; Supplementary Figure S1). By comparison, the AUCs for individual genes were 0.928 for HTR3C, 0.969 for RPS6KA6, and 0.836 for NETO2 (Figure 6D). At the optimal Youden-index-derived cutoff (≈0.73), the nomogram achieved sensitivity = 98.9%, specificity = 97.6%, positive predictive value (PPV) = 99.6%, and NPV = 93.2% (Supplementary Table S4). Internal calibration confirmed strong agreement between predicted and observed probabilities (Supplementary Figure S2). Specifically, the solid bias-corrected line closely tracks the 45-degree diagonal dashed line representing the ideal model, indicating that the risk probabilities of UC predicted by the nomogram are highly consistent with the actual diagnostic outcomes. This establishes the model’s high reliability for individualized probability estimation. DCA further showed that the nomogram provided greater net clinical benefit than either the “treat-all” or “treat-none” strategies across a wide range of threshold probabilities (Figure 6C). Across all training and validation cohorts, the nomogram curve remains substantially above the zero-benefit horizontal line (“None”) and the “treat-all” reference, demonstrating that the clinical implementation of this signature for UC screening would yield a significant net benefit by enhancing diagnostic precision and reducing unnecessary clinical workups. To evaluate generalizability, the model was externally validated in two independent cohorts. In the GSE48958 cohort, the nomogram yielded an AUC of 0.962, while the individual gene AUCs were 0.817 (HTR3C), 0.952 (RPS6KA6), and 0.740 (NETO2) (Figure 6G). The differential expression of all three genes was consistently observed in this cohort (Figure 6H), and DCA confirmed that the nomogram offered superior net benefit compared with both reference strategies within clinically relevant threshold ranges (Figure 6F). Similarly, in the GSE73661 validation cohort, the nomogram achieved an AUC of 0.985, with individual AUCs of 0.842 (HTR3C), 0.985 (RPS6KA6), and 0.766 (NETO2) (Figure 6J). The differential expression of the three key genes was again confirmed (Figure 6K), and the DCA results consistently demonstrated that the nomogram outperformed the “treat-all” and “treat-none” approaches in terms of net benefit (Figure 6I). Collectively, these results underscore the robust diagnostic performance and reproducibility of the three-gene signature across multiple datasets.

### 3.6. Identification of Shared Pathways and Key Signaling Pathways Through Integrated Multi-Omics Analysis

**Figure 7:**
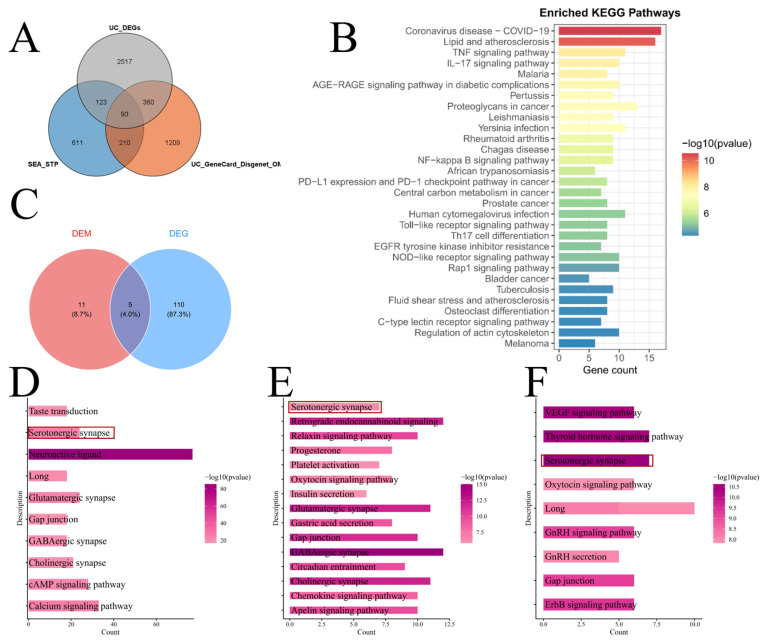
Identification of shared pathways and key signaling pathways through integrated multi-omics analysis. (**A**) Venn diagram showing the intersection of three gene sets. (**B**) KEGG pathway enrichment results for the candidate target genes. (**C**) Venn diagram illustrating the overlap between KEGG pathways enriched from differential metabolites and those from candidate target genes. (**D**) KEGG enrichment analysis of genes shared between the metabolite–target gene set and the NRRG gene set. (**E**) KEGG enrichment analysis of genes shared between UC DEGs and the NRRG gene set. (**F**) KEGG enrichment analysis of genes common to the metabolite –target gene set, UC-associated disease gene set, and NRRG gene set.

To further explore the potential functional roles of key NRRGs at the pathway level, we performed an integrated multi-omics analysis combining transcriptomic, metabolomic, and disease-associated gene datasets. First, 1872 UC-associated disease target genes were compiled from the OMIM, GeneCards, and DisGeNET databases (Supplementary Figure S3A). Metabolomic profiling identified 334 differential metabolites, for which approximately 10,500 and 9247 metabolite–target gene associations were predicted using the Similarity Ensemble Approach (SEA) and SwissTargetPrediction databases, respectively. After merging and deduplicating predictions from both sources, 1037 high-confidence metabolite-associated genes were retained. Intersecting these metabolite target genes (n = 1037) with transcriptomic differentially expressed genes (DEGs; n = 3093) and UC-associated disease genes (n = 1872) yielded a core set of 93 candidate target genes (Figure 7A). Supplementary Figure S3B–D illustrate the overlaps between the NRRG gene set and the metabolite target gene set, UC DEGs, and UC-associated disease gene set, respectively. These intersections highlight the concordance across data layers and provide a foundation for downstream enrichment analyses. KEGG pathway enrichment analysis of the 93 candidate genes revealed 115 significantly enriched pathways. The top 30 pathways were primarily linked to COVID-19, lipid metabolism and atherosclerosis, TNF/IL-17–mediated inflammatory signaling, and multiple cancer-related processes (Figure 7B). Separately, KEGG enrichment of the 334 differential metabolites identified 16 significantly altered metabolic pathways. Intersecting the enriched pathways from gene- and metabolite-level analyses uncovered five shared pathways: neuroactive ligand–receptor interaction, serotonergic synapse, prolactin signaling pathway, choline metabolism in cancer, and chemical carcinogenesis–receptor activation (Figure 7C). To further evaluate the involvement of NRRGs across data sources, additional intersection analyses were conducted. Overlap between the metabolite target gene set (n = 1037) and the NRRG gene set (n = 448) yielded 109 common genes, whose KEGG enrichment again highlighted the serotonergic synapse pathway (Figure 7D). Similarly, intersecting UC DEGs (n = 3093) with the NRRG gene set identified 50 overlapping genes, also significantly enriched in the serotonergic synapse pathway (Figure 7E). Moreover, a triple intersection of metabolite target genes, UC-associated disease genes (n = 1872), and NRRGs resulted in 12 shared genes, with KEGG enrichment consistently pinpointing the serotonergic synapse pathway as the top hit (Figure 7F). Collectively, these findings demonstrate that the serotonergic synapse pathway emerged repeatedly across multiple integration strategies and independent data layers, underscoring its robust and central role in the multi-omics landscape of ulcerative colitis.

### 3.7. Integrated Mapping of Differential Genes and Metabolites in the Key Signaling Pathway

**Figure 8:**
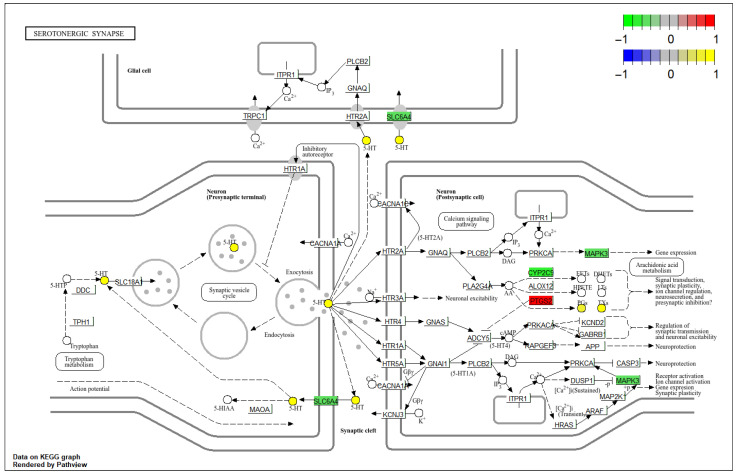
Integrated mapping of differentially expressed genes and differential metabolites in the serotonergic synapse pathway (hsa04726).

At the gene expression level, PTGS2 was significantly upregulated (log_2_FC = 2.42), whereas CYP2C9 (log_2_FC = −0.71), SLC6A4 (log_2_FC = −0.56), and MAPK3 (log_2_FC = −0.58) were downregulated. At the metabolite level, 11-dehydrothromboxane B_2_ (C05964, log_2_FC = 1.40), prostaglandin G_2_ (C05956, log_2_FC = 0.63), and serotonin (C00780, log_2_FC = 1.02) were all elevated, while DL-tryptophan (C00078, log_2_FC = −4.93) was markedly depleted (Figure 8). These coordinated changes in genes and metabolites within the same pathway reflect interconnected cross-omics alterations spanning precursor availability (DL-tryptophan depletion), transporter expression (SLC6A4 downregulation), neurotransmitter accumulation (serotonin increase), and downstream inflammatory signaling (PTGS2 upregulation and eicosanoid shifts), collectively illuminating key pathophysiological features of UC.

### 3.8. Differential Expression, Correlation, and Diagnostic Performance Analysis of Genes and Metabolites in the Key Signaling Pathway

**Figure 9:**
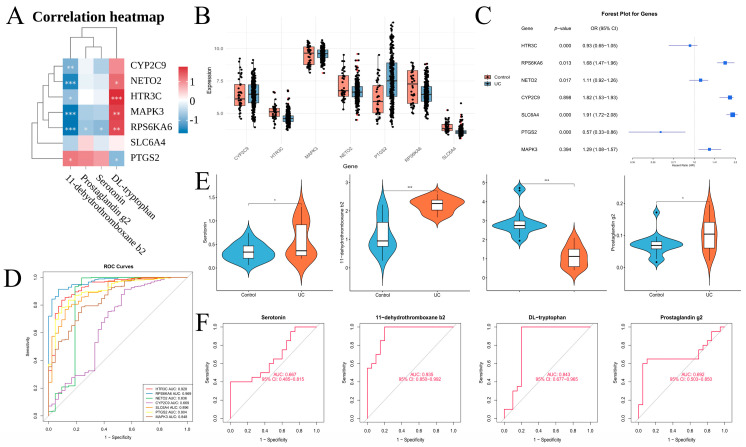
Differential expression, correlation structure, and discriminative performance of genes and metabolites associated with the 5-HT signaling pathway. (**A**) Spearman correlation heatmap showing gene–metabolite associations. (**B**) Differential expression of pathway-related genes and machine learning-identified key genes between UC and control groups. (**C**) Forest plot from univariate logistic regression analysis depicting associations between candidate genes and UC status. (**D**) ROC curves and corresponding AUC values for candidate genes. (**E**) Group-wise differences in pathway-related metabolites between UC and control groups. (**F**) ROC curves for pathway-related metabolites, with AUCs and 95% confidence intervals. * *p* < 0.05, ** *p* < 0.01, *** *p* < 0.001.

Based on the integrated mapping of the serotonergic synapse pathway (hsa04726), we further evaluated group-wise differences, gene–metabolite correlations, and the discriminative performance of pathway-related genes and differential metabolites (Figure 9A). Correlation heatmap analysis revealed distinct clustering patterns between genes and metabolites. Specifically, CYP2C9, NETO2, HTR3C, MAPK3, RPS6KA6, and SLC6A4 were generally negatively correlated with serotonin, prostaglandin G_2_, and 11-dehydrothromboxane B_2_, but positively correlated with DL-tryptophan. In contrast, PTGS2 showed opposite correlation patterns, reinforcing a coherent directional trend of molecular alterations within the pathway while also revealing nuanced stratification in the correlation structure. At the gene level, both pathway-related genes and the machine learning–identified key genes exhibited clear differential expression between UC and control groups (Figure 9B). Univariate logistic regression analyses, adjusted for age, assessed the association of each candidate gene with UC status, with results presented as forest plots (Figure 9C). ROC curve analysis indicated that most of these genes demonstrated measurable diagnostic performance: RPS6KA6, HTR3C, PTGS2, and SLC6A4 achieved AUCs of 0.969, 0.928, 0.904, and 0.896, respectively; MAPK3 and NETO2 yielded AUCs of 0.848 and 0.836; and CYP2C9 showed modest discriminative ability (AUC = 0.669) (Figure 9D). At the metabolite level, serotonin, DL-tryptophan, 11-dehydrothromboxane B_2_, and prostaglandin G_2_ all differed significantly between UC patients and controls (Figure 9E). Corresponding ROC analyses revealed AUCs of 0.935 for 11-dehydrothromboxane B_2_, 0.843 for DL-tryptophan, 0.692 for prostaglandin G_2_, and 0.667 for serotonin (Figure 9F). Together, these results demonstrate consistent signals across correlation architecture and diagnostic performance among serotonergic synapse-related genes and metabolites, offering complementary molecular features that enhance the characterization of UC-associated phenotypes.

### 3.9. RT-qPCR Validation

**Figure 10:**
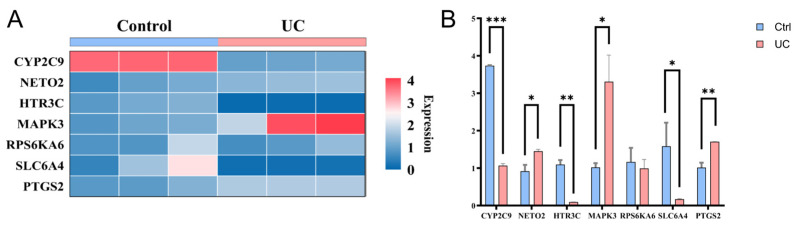
RT-qPCR validation of hub genes. (**A**) Heatmap showing expression profiles of hub genes across the two study groups. (**B**) Relative expression levels of hub genes in peripheral blood samples from UC patients compared with healthy controls. * *p* < 0.05, ** *p* < 0.01, *** *p* < 0.001.

To validate the differential expression of genes in the serotonergic synapse (5-HT signaling) pathway identified in prior transcriptomic analyses, we performed reverse transcription quantitative polymerase chain reaction (RT-qPCR) on peripheral blood mononuclear cells (PBMCs) isolated from patients with UC and age-matched healthy controls. Seven genes were selected based on their relevance to the pathway and their differential expression patterns: the machine learning identified key genes NETO2, RPS6KA6, and HTR3C, along with CYP2C9, MAPK3, SLC6A4, and PTGS2, all of which emerged from the serotonergic synapse pathway enrichment analysis. Hierarchical clustering of normalized RT-qPCR expression data clearly separated UC samples from controls, with upregulated genes shown in red and downregulated genes in blue (Figure 10A). Quantitative analysis confirmed significant expression changes for most genes (Figure 10B). Compared with healthy controls, UC patients exhibited the significant upregulation of CYP2C9 (*p* < 0.01), MAPK3 (*p* < 0.01), and PTGS2 (*p* < 0.01), and the significant downregulation of NETO2 (*p* < 0.01), HTR3C (*p* < 0.01), and SLC6A4 (*p* < 0.05). RPS6KA6 showed a consistent downward trend but did not reach statistical significance (*p* > 0.05). These results independently corroborate the dysregulation of the serotonergic signaling pathway in UC and highlight the potential of these genes, particularly the statistically validated ones, as candidate biomarkers for disease monitoring or as targets for therapeutic intervention.

## 4. Discussion

This study integrates high-throughput serum metabolomics with large-scale colonic mucosal transcriptomics to provide the first systematic characterization of perturbations in the serotonergic synapse pathway in UC from an integrative “systemic metabolism–local gene expression” perspective. Our findings not only confirm that this pathway functions as a critical hub linking systemic metabolic shifts to local intestinal inflammation, but also demonstrate that machine learning algorithms successfully pinpointed three key neurotransmitter receptor-related genes, HTR3C, RPS6KA6, and NETO2. A diagnostic nomogram built on these three genes exhibited consistent discriminative performance across multiple independent cohorts, achieving AUC values exceeding 0.96. Experimental validation further confirmed statistically significant differential expression for HTR3C and NETO2. At this stage, this three-gene panel should be considered a candidate biomarker signature rather than a clinically validated diagnostic tool. These results provide preliminary molecular evidence to support the development of noninvasive diagnostic strategies for UC, while offering deeper mechanistic insights into the gut–brain axis. Nonetheless, the reported AUC values, particularly the near-perfect performance in the training set (~0.998), must be interpreted with caution. In a heterogeneous disease such as UC, such high diagnostic accuracy may reflect residual overfitting or optimism bias inherent to retrospective datasets, despite our efforts to maintain strict cohort separation. Furthermore, the absence of nested cross-validation or repeated resampling in the current study is a recognized limitation that may affect the assessment of model stability across broader populations. Moreover, RT-qPCR validation of serotonergic synapse pathway genes in an independent peripheral blood cohort showed directional consistency with the cross-omics integration: PTGS2 and MAPK3 were upregulated, whereas CYP2C9, NETO2, HTR3C, and SLC6A4 were downregulated. However, due to the limited sample size of this validation cohort, these observations warrant confirmation in larger, prospective studies.

One of the most prominent findings of this study is the consistent enrichment of the serotonergic synapse pathway at both metabolomic and transcriptomic levels, revealing a distinctive neuro–immune–metabolic interaction network underlying UC pathology. The observed metabolomic alterations may largely reflect downstream consequences of transcriptional regulation [32]; however, metabolic shifts can also feed back to influence gene expression, for example, through serotonin modulating inflammatory pathways via receptor signaling or epigenetic mechanisms [33]. This bidirectional crosstalk strengthens the biological plausibility of our multi-omics observations, although establishing causal directionality will require dedicated mechanistic studies. We observed a marked reduction in serum tryptophan levels alongside elevated serotonin (5-hydroxytryptamine, 5-HT), which closely paralleled the significant downregulation of the serotonin transporter SLC6A4 in colonic tissues. These findings support impaired 5-HT reuptake as a plausible mechanism: intestinal inflammation appears to suppress the epithelial expression of SERT, the protein encoded by SLC6A4, leading to the extracellular accumulation of 5-HT [34]. Prior studies have shown that reduced SLC6A4 activity results in excessive 5-HT buildup in synaptic and mucosal compartments, thereby amplifying 5-HT receptor-mediated signaling in immune cells and promoting the release of pro-inflammatory cytokines [35,36]. Accordingly, the coordinated pattern observed here, tryptophan depletion, SLC6A4 downregulation, and serotonin accumulation, forms a biologically coherent and testable pathological loop that bridges systemic metabolic dysfunction and local tissue injury. Specifically, systemic tryptophan depletion reflects a massive metabolic shunt toward the serotonergic pathway, where the subsequent failure of SLC6A4-mediated reuptake leads to a localized “serotonin storm” within the mucosal compartment. This excess serotonin, coupled with the identified remodeling of HTR3C, RPS6KA6, and NETO2, provides a sustained mechanistic foundation for the PTGS2-driven inflammatory cascade, illuminating how neuro-endocrine dysregulation drives the chronic relapsing nature of UC. This framework not only reconciles the concordant metabolic and transcriptional alterations but also provides a clear trajectory for future mechanistic validation.

Notably, serotonergic dysregulation does not occur in isolation. It coincides with the amplification of inflammatory lipid mediator networks. We observed the significant upregulation of PTGS2 (cyclooxygenase-2), along with elevated levels of prostaglandin G_2_ and 11-dehydrothromboxane B_2_, indicating active engagement of the arachidonic acid cascade [37]. Beyond its role as a pro-inflammatory neurotransmitter, excess 5-HT may further induce PTGS2 expression through positive feedback mechanisms. This drives prostaglandin synthesis and intensifies inflammatory cascades, consistent with the increased prostaglandin G_2_ and thromboxane metabolites detected in this study [38]. Moreover, prior evidence indicates that tryptophan metabolism can be diverted into alternative branches, such as the kynurenine and indole pathways, which in turn modulate mucosal immune homeostasis and epithelial repair programs [39]. These findings align conceptually with the metabolic–transcriptional coupling we identified and reinforce the view that serotonergic signaling occupies a central node in UC-associated neuroimmune regulation. Importantly, whereas earlier studies focused predominantly on local tissue alterations, our multi-omics data demonstrate that these localized signaling disruptions are sufficient to generate detectable systemic metabolic signatures. This observation underscores the therapeutic potential of targeting serotonergic pathways to interrupt the self-sustaining inflammatory cycle in UC.

The three-gene signature derived from the NRRG pool, HTR3C, RPS6KA6, and NETO2, demonstrates both biological plausibility and translational relevance. Compared with prior multi-omics diagnostic models that rely on broad metabolic panels (e.g., lipid species or tricarboxylic acid cycle intermediates), our NRRG-focused signature offers distinct specificity by capturing dysregulated neuroendocrine signaling. For instance, while Janker et al. [25] highlighted the importance of mucosal protein profiles in clinical remission, our findings uniquely reveal a “full-chain” dysregulation of the serotonergic synapse, spanning from precursor tryptophan depletion in the serum to specific receptor remodeling in the mucosa. This cross-layer consistency provides a higher degree of mechanistic coherence than models built on fragmented omics correlations, positioning our signature as a distinctive tool for characterizing the neuro-epithelial microenvironment in UC. HTR3C, which encodes an ionotropic 5-HT_3_ receptor subunit, emerged as a stable feature across all machine learning frameworks. In UC pathophysiology, HTR3C acts as more than a marker; it is a critical transducer at the neuro-epithelial interface. Its downregulation likely disrupts the rapidionflux required for synchronized enterochromaffin cell-neuron signaling, directly contributing to the visceral hypersensitivity and dysregulated mucus secretion that characterize active colitis. This finding suggests that UC-associated neuroendocrine imbalance extends beyond altered neurotransmitter distribution to include remodeling of receptor sensitivity and signal transduction [40,41] mechanisms, consistent with prior reports linking 5-HT_3_ receptors to the regulation of intestinal motility and secretion [42,43]. RPS6KA6, a member of the ribosomal S6 kinase (RSK) family that functions downstream of the MAPK/ERK pathway [44,45], has been extensively investigated in oncology but remains largely unexplored in inflammatory bowel disease. We propose that RPS6KA6 functions as a molecular “rheostat” for mucosal homeostasis; its altered expression may compromise the epithelial ability to suppress NF-κB-dependent pro-inflammatory signals under chronic oxidative stress, thereby exacerbating cell apoptosis and mucosal ulceration [46]. NETO2, an auxiliary subunit best known for its role in regulating glutamate receptor function and synaptic transmission [47]. By stabilizing receptor assemblies at synapse-like neuro-epithelial junctions, NETO2 potentially modulates epithelial barrier permeability and localized immune cell recruitment, suggesting that UC involves a fundamental reorganization of how the intestinal mucosa senses and responds to neuro-endocrine stimuli [48,49]. Critically, the nomogram incorporating these three genes consistently achieved high discriminative performance in two independent external cohorts (AUC > 0.96), surpassing several existing single-biomarker approaches [50]. Decision curve analysis further confirmed a clear net clinical benefit, indicating that neuroendocrine-informed features provide independent diagnostic value. Nevertheless, since this study did not perform a head-to-head comparison with established clinical biomarkers such as C-reactive protein (CRP) or fecal calprotectin, the incremental benefit of this signature over existing standard-of-care tests remains to be quantified. Future prospective studies are necessary to evaluate whether this multi-omics approach can enhance or replace current clinical diagnostic workflows.

Despite these strengths, several limitations warrant acknowledgment. First, although our findings were supported by external validation, this study is retrospective in design; thus, large prospective clinical cohorts are still needed to assess the dynamic utility of the proposed model for early diagnosis and disease monitoring. A primary limitation of this study is the unaddressed impact of clinical heterogeneity and missing metadata across the included cohorts. Integrating serum metabolomic data from our cohort with transcriptomic profiles from independent GEO datasets, derived from distinct patient populations, raises valid concerns about potential biological bias. Specifically, the absence of sex data in key training datasets is a significant limitation, as tryptophan metabolism and serotonergic signaling are known to exhibit sexual dimorphism. Furthermore, varying treatment exposures (such as 5-aminosalicylates, corticosteroids, or biologics like infliximab) and differences in disease stage across cohorts can profoundly modulate both mucosal gene expression and systemic metabolic profiles. These confounding factors may bias our findings toward specific inflammatory states, and the correlations observed between tryptophan depletion and transporter downregulation may be partially influenced by therapy-induced metabolic shifts rather than disease pathology alone. While gene–metabolite associations were inferred through pathway-level intersection and further supported by RT-qPCR validation in peripheral blood, these relationships remain correlational and may be confounded by inter-cohort heterogeneity. Although multi-algorithm feature selection and validation in multiple external cohorts enhanced reliability, future studies would benefit substantially from paired multi-omics measurements obtained from the same individuals. Second, while we delineate molecular alterations within the serotonergic synapse pathway, causal evidence linking RPS6KA6 and NETO2 to the maintenance of intestinal barrier integrity is currently lacking. Functional validation using appropriate animal models is therefore warranted. Finally, serum metabolomic profiles are inherently susceptible to dietary and pharmacological influences. Although patients with severe comorbidities were excluded, future investigations should implement stricter control of lifestyle-related variables. Ongoing and planned work will focus on the functional characterization of these key genes in human intestinal organoid systems and on exploring therapeutic strategies targeting serotonergic metabolism, such as tryptophan supplementation or selective receptor modulation, to evaluate their potential in modulating UC pathophysiology.

## 5. Conclusions

This study integrates serum LC–MS metabolomics with multicenter colonic mucosal transcriptomics to reveal significant systemic co-remodeling in UC at the “metabotype–gene transcription” interface. Through multidimensional data convergence, we identified a neuroendocrine regulatory network centered on three key pathways: neuroactive ligand–receptor interactions, the serotonergic synapse, and prolactin signaling, highlighting tight coupling between neurotransmitter/hormonal signals and immune-inflammatory mediators. Notably, within the serotonergic synapse pathway, we observed a coordinated, full-chain dysregulation encompassing precursor metabolism (decreased tryptophan), neurotransmitter accumulation (elevated 5-HT), transporter suppression (SLC6A4 downregulation), and enhanced production of downstream inflammatory mediators (PTGS2 upregulation). This cascade provides highly consistent molecular support for the “brain–gut–microenvironment” imbalance central to UC pathophysiology. Building on this framework, we leveraged the NRRG pool and applied an ensemble of machine learning algorithms, LASSO, random forest, and SVM-RFE, to identify and validate a three-gene signature comprising HTR3C, RPS6KA6, and NETO2. The resulting nomogram model demonstrated excellent diagnostic performance, achieving AUC values exceeding 0.96 in the training set and maintaining robust accuracy across two independent external cohorts. Decision curve analysis confirmed a clear net clinical benefit. Experimental validation further verified statistically significant differential expression for HTR3C and NETO2. Moreover, RT-qPCR in an independent peripheral blood cohort, albeit small, replicated the upregulation of PTGS2 and MAPK3 alongside downregulation of CYP2C9, NETO2, HTR3C, and SLC6A4, reinforcing the reproducibility of serotonergic synapse pathway perturbations beyond tissue-specific contexts. Collectively, this work delivers preliminary multi-omics evidence supporting the identification of a candidate biomarker signature. The primary distinctiveness of this study lies in its shift from broad inflammatory profiling toward a targeted analysis of the neuro–immune–metabolic axis via NRRGs. By demonstrating the systemic-to-local consistency of serotonergic pathway perturbations, we provide a more focused biological interpretation than previous multi-omics studies, facilitating the development of neuroendocrine-informed diagnostic strategies in UC. While promising, this model requires rigorous testing in real-world diagnostic scenarios and comparison with traditional inflammatory markers before it can be considered for clinical implementation. Nevertheless, larger prospective cohorts and functional studies are needed to confirm the robustness and biological significance of these findings.

## Figures and Tables

**Table 1:** Summary of datasets used in this study.

Dataset	GPL Platform	UC	HC	Sample Type	Age Range (Years)	Disease Severity	Treatment Status
GSE87473	GPL13158	106	21	Colon mucosal biopsy	6–77	Extensive/Limited Colitis	Screening (Baseline)
GSE92415	GPL13158	162	21	Colon mucosal biopsy	19–77	Mayo score (0–12)	Baseline (Pre-treatment)
GSE48958	GPL6244	13	8	Colon mucosal biopsy	Not reported	Active/Inactive UC	Baseline status
GSE73661	GPL6244	67	12	Colon mucosal biopsy	Not reported	Mayo score (0–12)	Pre/Post IFX or VDZ therapy

**Table 2:** Baseline characteristics of study participants.

Variables	*n* ^#^	Overall *n* = 60 *	NOR *n* = 30 *	UC *n* = 30 *	*p*-Value ^1^
Gender	60				0.602
Female		26 (43%)	14 (47%)	12 (40%)	NA
Male		34 (57%)	16 (53%)	18 (60%)	NA
MCC	30				1.000
E1		3 (10%)	0 (NA%)	3 (10%)	NA
E2		8 (27%)	0 (NA%)	8 (27%)	NA
E3		19 (63%)	0 (NA%)	19 (63%)	NA
NA		30	30	0	NA
Age	60	35 ± 11	30 ± 6	41 ± 12	<0.001
BMI	60	22.65 ± 3.03	22.89 ± 2.21	22.40 ± 3.69	0.536
Mayo Score	30	6 ± 2	NA ± NA	6 ± 2	NA
NA		30	30	0	NA
CP	60	144 ± 174	13 ± 12	274 ± 162	<0.001
CRP	60	20 ± 38	3 ± 3	37 ± 48	<0.001
ESR	60	9 ± 11	4 ± 2	15 ± 14	<0.001
HB	60	127 ± 17	132 ± 6	121 ± 22	0.009

Note: Categorical variables were analyzed using the Pearson chi-square test or Fisher’s exact test, as appropriate. Continuous variables were compared using Welch’s two-sample *t*-test. When data were not comparable or statistical tests were not applicable, *p* values were reported as NA. NOR denotes the healthy control group; UC, the ulcerative colitis group; BMI, body mass index; CP, fecal calprotectin; CRP, C-reactive protein; ESR, erythrocyte sedimentation rate; HB, hemoglobin; and MCC, the Mayo endoscopic subscore. ^#^ *n* Non-missing. * *n* (%); Mean ± SD. ^1^ Pearson’s Chi-squared test; Fisher’s exact test; Welch Two Sample *t*-test; NA.

## Data Availability

The datasets presented in this study are available in online repositories. The names of the repository/repositories and accession number(s) are provided in the article/Supplementary Materials. Additional data supporting the conclusions of this study will be made available by the authors upon reasonable request.

## Supplementary Materials

The following supporting information can be downloaded at: https://www.mdpi.com/article/10.3390/metabo16040263/s1, Figure S1: ROC curve analysis of the nomogram in the training set; Figure S2:Internal calibration curve of the nomogram; Figure S3: Integration and overlap analysis of UC-associated gene sets and NRRGs; Table S1: List of the 448 NRRGs; Table S2: Primer sequences used for RT-qPCR; Table S3: Top 20 candidate DEGs and metabolites; Table S4: Nomogram train ROC metrics.
