# Supplementary material for: Integrated Metabolomic and Transcriptomic Analyses Reveal Alterations in the Serotonergic Synapse Pathway and a Robust Diagnostic Model in Ulcerative Colitis
Source: Metabolites. 2026 Apr 14;16(4):263. doi: 10.3390/metabo16040263 (PMC13117237; doi:10.3390/metabo16040263)
Supplement: Supplementary file 1 [file metabolites-16-00263-s001.zip › Certificate_of_Editing_Service.pdf]

MedSci

Editing By Professional Editors

CERTIFICATE OF ENGLISH EDITING

This document certifies that the manuscript entitled "Integrated Metabolomic and Transcriptomic Analyses Reveal Alterations in the Serotonergic Synapse Pathway and a Robust Diagnostic Model in Ulcerative Colitis" was proofread and edited for proper English language, grammar, punctuation, spelling, and overall style by one or more of the qualified scientific editors at MedSci, all of whom are native English speakers. Neither the research content nor the authors' intentions were altered in any way during the editing process.

Documents receiving this certification should be English-ready for publication; however, the author can accept or reject our suggestions and changes. To see the final MedSci edited version, please visit our verification page. If you have any questions or concerns about this document or certification, please contact us at editing@medsci.cn.

First Author:Haiyan Wang

DATE:2026-03-18

SIGNATURE: MedSci

CODE:0318-E458-2CD9-5B5F-DB67

This certificate may be verified at

https://editing.medscihealthcare.com/dist/medsci-order/#/verify
