# Supplementary figures and images for: Integrated Metabolomic and Transcriptomic Analyses Reveal Alterations in the Serotonergic Synapse Pathway and a Robust Diagnostic Model in Ulcerative Colitis
Source: Metabolites. 2026 Apr 14;16(4):263. doi: 10.3390/metabo16040263 (PMC13117237; doi:10.3390/metabo16040263)

**Train set (Nomogram with 95% CI)**

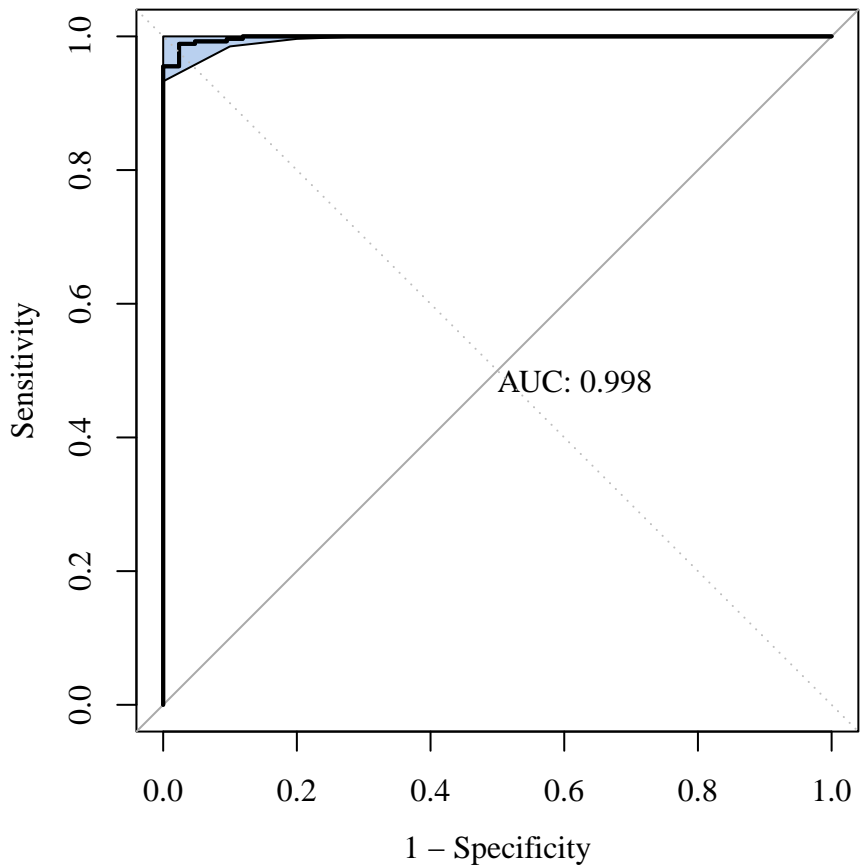

Supplement: Supplementary file 1 [file metabolites-16-00263-s001.zip › Supplementary Figure S1.pdf]

Observed Probability

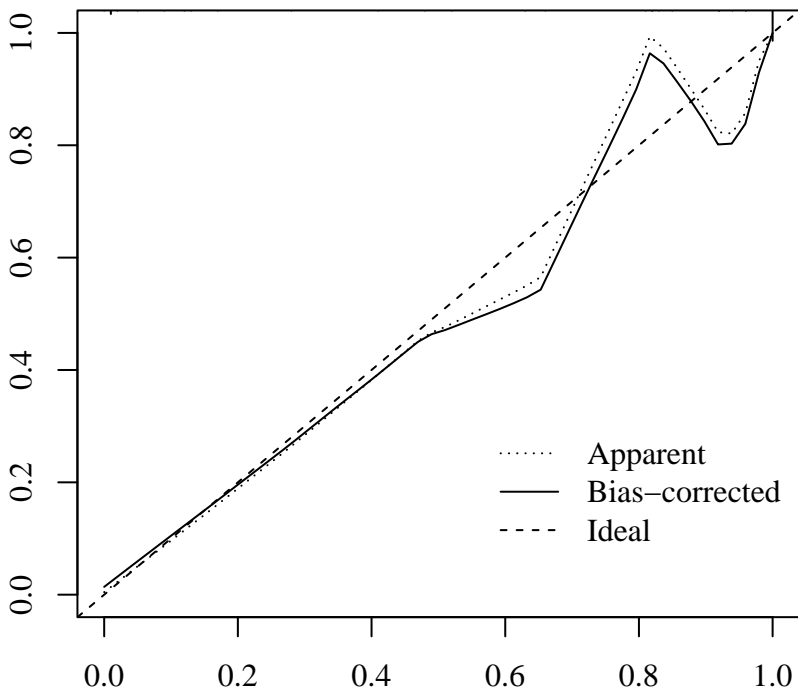

Predicted Probability

Supplement: Supplementary file 1 [file metabolites-16-00263-s001.zip › Supplementary Figure S2.pdf]

A

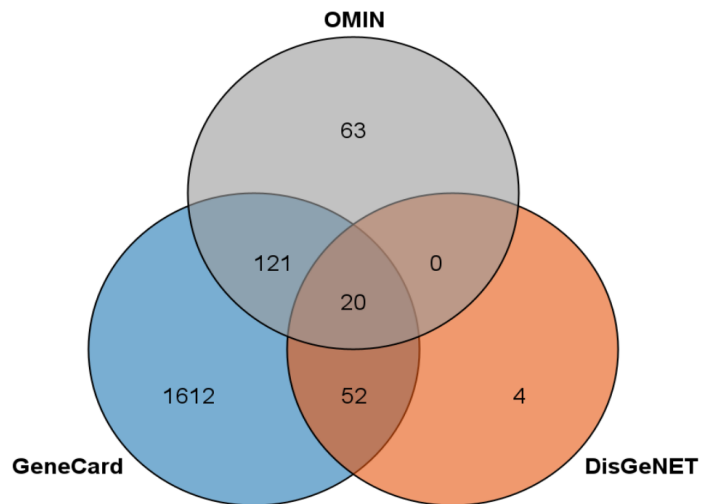

B

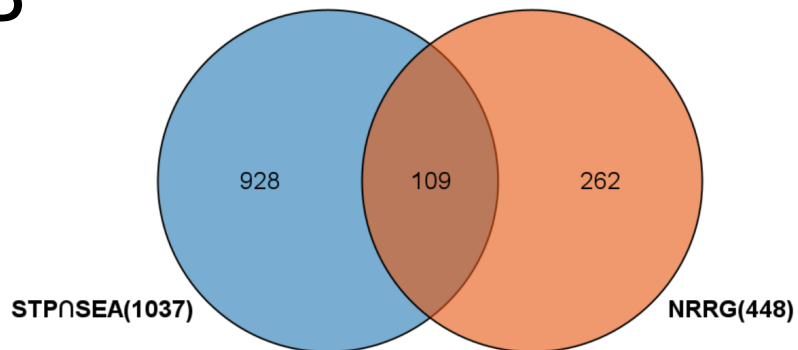

C

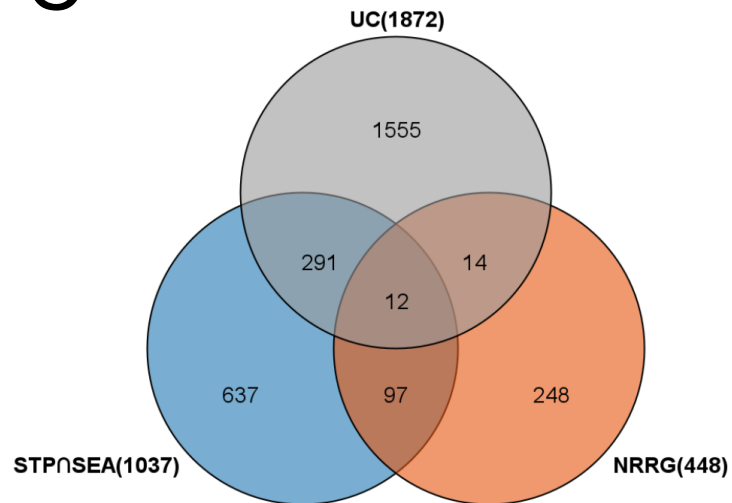

D

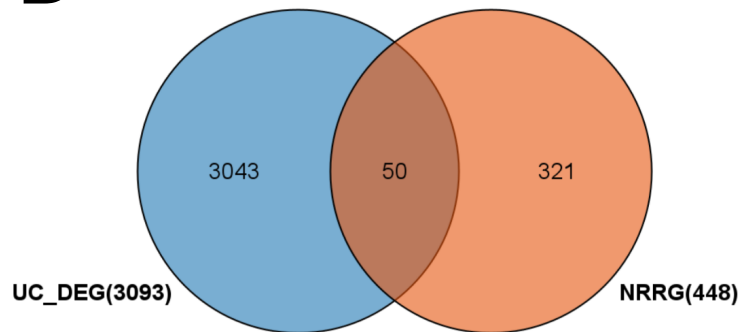

Supplement: Supplementary file 1 [file metabolites-16-00263-s001.zip › Supplementary Figure S3.pdf]
